# Comparative Effects of Neuromuscular- and Strength-Training Protocols on Pathomechanical, Sensory-Perceptual, and Motor-Behavioral Impairments in Patients with Chronic Ankle Instability: Randomized Controlled Trial

**DOI:** 10.3390/healthcare10081364

**Published:** 2022-07-22

**Authors:** Kyung-Min Kim, Alejandro Estepa-Gallego, María D. Estudillo-Martínez, Yolanda Castellote-Caballero, David Cruz-Díaz

**Affiliations:** 1 Department of Sport Science, Sungkyunkwan University, Suwon-si 16419, Gyeonggi-do, Korea; km.kim@g.skku.edu; 2 Department of Kinesiology and Sport Sciences, University of Miami, Coral Gables, FL 33146, USA; 3 Department of Health Sciences, Faculty of Health Sciences, University of Jaén, E-23071 Jaén, Spain; alejandroestepa@thefisioprogram.com (A.E.-G.); dcruz@ujaen.es (D.C.-D.); 4 Department of Statistics, Faculty of Experimental Sciences, University of Jaén, E-23071 Jaén, Spain; mdestudi@ujaen.es; 5 Department of Physical Activity and Sport, Faculty of Sport Sciences, Campus of Excellence Mare Nostrum, Universidad de Murcia, E-30720 Murcia, Spain

**Keywords:** chronic ankle instability, neuromuscular training, strength training, self-reported instability, dynamic balance

## Abstract

(1) Background: Chronic ankle instability (CAI) is a complex condition that includes limited mobility, perceived instability, and recurrent ankle sprains are common characteristics that reduce the quality of life in subjects who suffer from CAI. Neuromuscular training and strength training have been recommended in CAI management interventions. However, there are contradictory findings on results when comparing neuromuscular training, strength training, and the control group. The objective of this study was to compare the effectiveness of 8 weeks of neuromuscular intervention training, strength training, and no intervention in a sporting population with reported CAI. (2) Methods: Sixty-seven athletes with CAI were randomly assigned to a neuromuscular training group (NG), strength training group (SG), or control group (CG). Participants completed 8 weeks of neuromuscular training (a combination of static and dynamic exercises), strength training (resistance band exercises), or no training. Outcome measures were assessed at baseline and after 8 weeks and included selfs-reported instability feeling (CAIT), dynamic balance (SEBT), ankle dorsiflexion range of motion (WBLT), and functional status (FAAM and FAAM-SPORT). (3) Results: There were significant differences between strength and control groups in the posteromedial direction of SEBT, FAAM, and FAAM-SPORT after 8 weeks of intervention. (4) Conclusions: Neuromuscular training and strength training based on resistance bands exercises showed significant improvements in ankle dorsiflexion, subjective feeling of instability, functional status, and dynamic balance in patients with CAI.

## 1. Introduction

An ankle sprain is the major ankle injury in sport, with an incidence of 76.7% of ankle injuries [1] and an estimated prevalence of ankle injuries of 11.88%. Only 50% sought professional medical help, so it is plausible that the incidence of ankle sprains may be higher than described in the literature [2]. Around 50% of ankle sprains occur during sports practice, more frequently in indoor sports such as volleyball and basketball [1]. It is also common in soccer since around 58.5% of soccer and basketball players have suffered at least one ankle sprain in their entire career [1,2,3,4]. There is a high economic impact related to ankle sprains: the cost ranges from USD 292 to USD 2268 for each ankle sprain [5].

An initial ankle sprain is the principal risk factor for developing chronic ankle instability (CAI), with a prevalence ranging from 0% to 73% [2,4]. The term CAI is defined as the inability to maintain the normal mobility of the ankle, losing its control in some situations and causing repetitive sprains and constant unstable feeling of the ankle joint during the execution of functional activities [4]. In 2019, Hertel et al. [4] proposed an updated model of CAI as more evidence became available in the literature that evolved from the original model in 2002 [6]. The updated model was developed based on dynamic complex systems [7] and Melzack’s theory of pain neuromatrix [8]. With the interactions of pathomechanics, sensory–perceptual, and motor-behavioral impairments, it is possible to see various patient scenarios with unique combinations of impairment-related symptoms. However, empirical evidence suggests that patients with CAI commonly present with limited dorsiflexion range of motion (DFROM), impaired postural control, self-reported ankle dysfunction, and perceived joint instability [2,4]. Regarding DFROM, there is no consensus establishing a cut-off point or normative value between CAI and copers due to the influence of anthropometric characteristics, so the comparison between the affected and unaffected sides could be clinically useful [1,2,3,4].

A large body of evidence suggests that neuromuscular training or strength training is an effective intervention to improve postural control [9,10], functional status [11], DFROM [12], and perceived joint instability [9,10] in patients with ankle sprains and CAI. From the clinical standpoint, it is interesting to see which intervention is more effective as clinicians always look for the best intervention. However, few studies directly compare these two promising interventions for CAI. To the best of our knowledge, only one research group examined the comparative effects of balance- and strength-training protocols regarding their impact on clinical and patient-reported outcomes [13,14]. Hall et al. [13,14] reported that both protocols are effective but failed to determine the superiority of one intervention over the other and concluded that both protocols are equally effective. However, by inspecting individual results, the specificity of each intervention may exist: the eccentric strength of eversion following the strength-training protocol was greater while Balance Error Scoring System scores were lower following the balance-training protocol compared with the control (no active exercise) protocol, but was not with the strength training protocol. The lack of differences in training protocol may be due to the small sample size (*n* = 13) and length of intervention (6 weeks). Thus, a follow-up study with a larger sample size and longer duration of the intervention could be a benefit in determining the superiority between neuromuscular training (NTP) and strength-training protocols (STP).

We hypothesized that both intervention groups would obtain significant improvements, with a possible superiority of NTP over STP because it is an integral treatment that includes more elements susceptible to being affected in patients with CAI. Theoretically, patients with CAI may present with a unique set of symptoms resulting from the interactions of the pathomechanics, sensory–perceptual, and motor-behavioral impairments that are also influenced by personal and environmental factors [15]. In order to determine the efficacy of two interventions for CAI in a single study, we are inherently limited to selecting several outcome variables that are common in CAI training studies, such as limited DFROM, impaired postural control, self-reported ankle dysfunction, and perceived joint instability [2,4]. Thus, the purpose of the present study was to compare the efficacy of NTP and STP on pathomechanics (limited DFROM), sensory–perceptual (self-reported ankle dysfunction and perceived instability), and motor-behavioral impairments (postural control) in patients with CAI.

## 2. Materials and Methods

### 2.1. Participants

Participants in a sporting population between 18 and 60 years were recruited via social media, posters in different university locations, and by word of mouth. They were enrolled if they met the inclusion criteria. Inclusion criteria were consistent with recommendations made by International Ankle Consortium [16] and were checked before the intervention: (1) to have suffered the first sprain at least 6 months before the beginning of the study; (2) participants could not have suffered an acute sprain in the 6 previous weeks to the beginning of the study; (3) ankle surgery in the last 3 months; (4) epilepsy or previous seizures; (5) not lower limb nor another known injury that affects the sensory–motor function; (6) participants with bilateral ankle dysfunction could participate, but the ankle enrolled was the most injured; (7) CAIT score ≤ 24; (8) finally, we also added another inclusion criteria for our investigation—to be physically active (perform physical activity at least 3 h per week). Exclusion criteria were: to be receiving treatment for the ankle in the 6 weeks before our intervention; to have suffered an acute sprain in the 6 weeks before the intervention; history of other lower-extremity injuries or neuromuscular deficits different to CAI; a previous recent surgical intervention that affects the lower limb.

Enrolled participants were randomly assigned to one of 3 groups: neuromuscular training group (NG), strength training group (SG), and control (no intervention) group (CG). The intervention sequence was randomized by an independent investigator who used a random number generator and sealed the treatment sequence in opaque envelopes which were opened before performing the first intervention.

This study was approved by the University of Jaen’s Human Ethical Committee and registered as NCT05250739 on Clinicaltrial.gov. The intervention was conducted following the Declaration of Helsinki, good clinical practices, and applicable laws and regulations and met the CONSORT guidelines standards [17]. We obtained Informed Consent from all participants, and all their rights were protected.

### 2.2. Intervention

All participants underwent eight weeks of an intervention program in their assigned groups. Participants allocated to experimental groups were instructed to be focused on maintaining a proper and controlled form during the exercise performance. Although the pain was not initially included as a study variable, participants were told to avoid pain during exercise performance by adapting the level of difficulty of the exercises.

#### 2.2.1. Control Group (CG)

Participants in this group received no intervention during the 8 weeks.

#### 2.2.2. Neuromuscular Training Group (NG)

**Figure 1:**
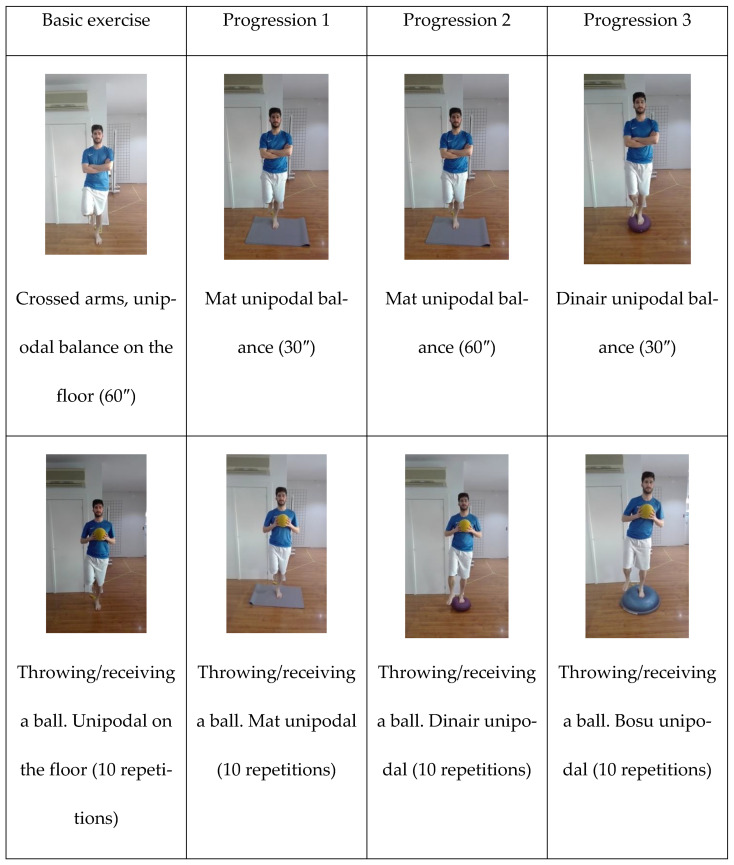

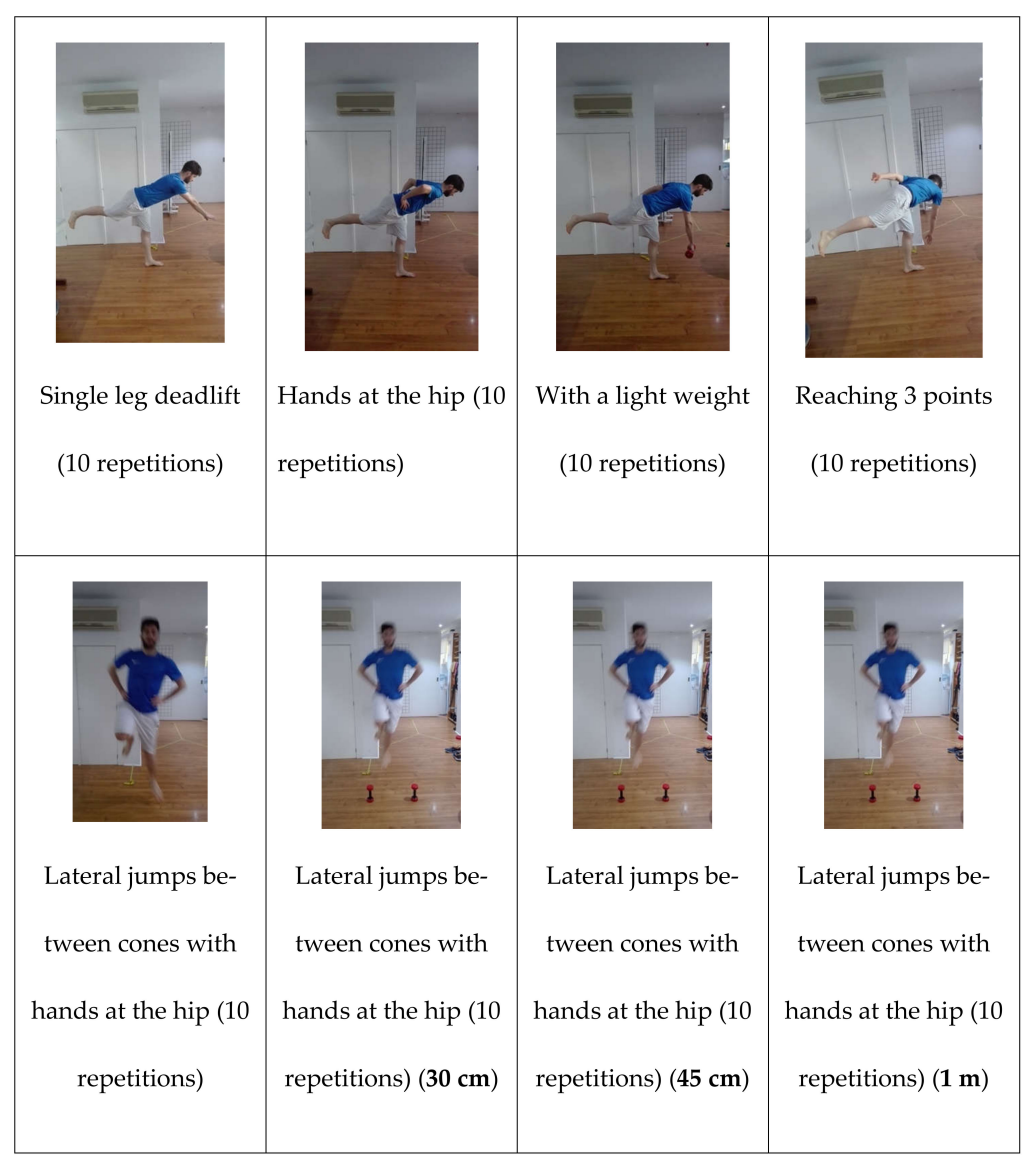

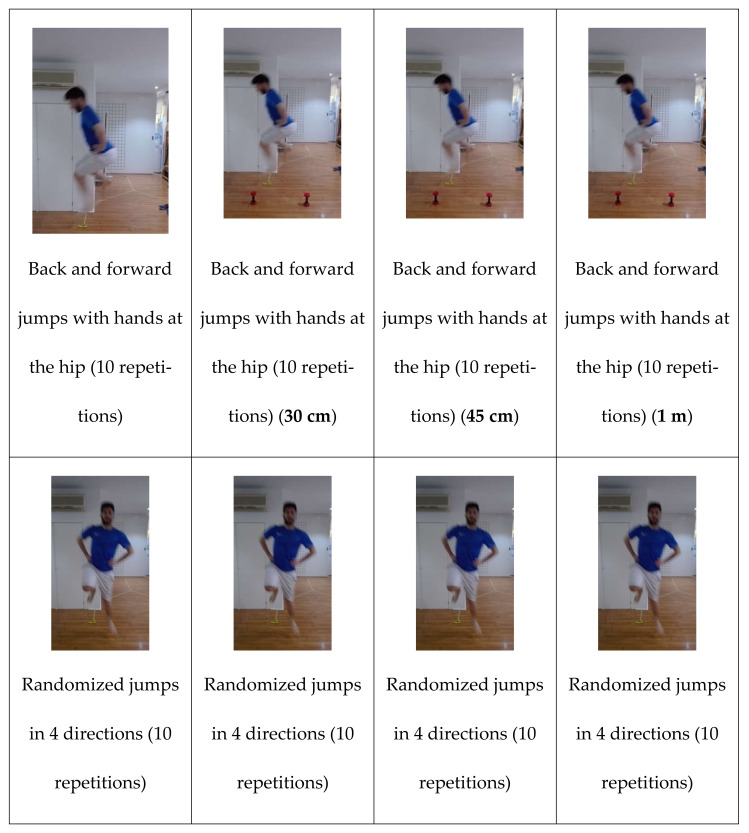
Neuromuscular exercises.

Patients allocated to NG completed 16 training sessions divided into 8 weeks. The intervention consisted of 6 neuromuscular exercises, increasing the difficulty progressively as the participants controlled the execution, and they did not start the next progression until performing a complete circuit in the level before with proficiency (Figure 1). Participants in this group performed all exercises on their barefoot with the CAI limb. In order to achieve a correct progression in the exercises, the level of difficulty was increased, different support bases were used by the patient (double support vs. balance on one leg), the type of support surface was made progressively more difficult to keep stable due to the nature of the instrument used during the exercise performance (stable floor, mat, dynair, or busu), the complexity of the task was altered, and external materials that suggest a handicap for the maintenance of the balance were used.

#### 2.2.3. Strength Training Group (SG)

**Figure 2:**
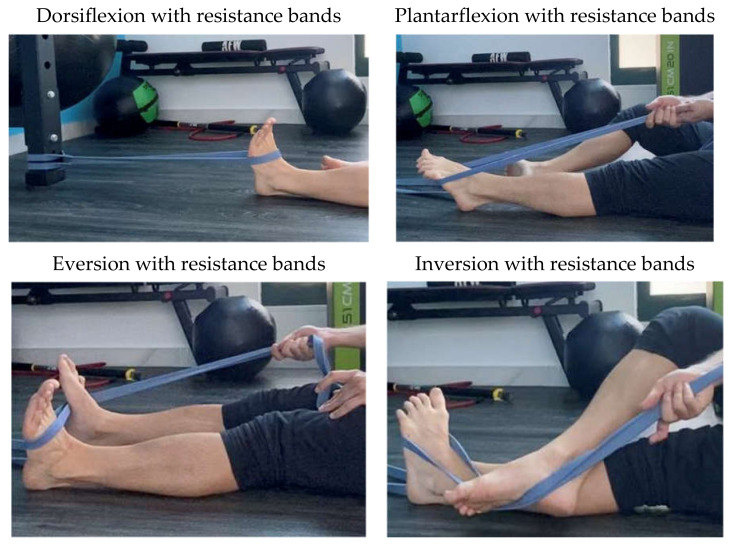
Strength training exercises.

Participants in this group performed ankle exercises with resistance bands (Figure 2). In order to perform the proposed exercises, the patients had to place the theraband around the foot of the affected ankle. They were instructed to tie the elastic band to a firm surface or to use the contralateral side as a support point while performing inversion, eversion, dorsiflexion, and plantarflexion exercises. Participants were asked to focus on the complete range of motion with a proper form avoiding compensatory movements and the presence of pain. Although elastic bands make it difficult to quantify the force production, the researcher tried to achieve homogeneous training among the participants, indicating that they should perform the exercises with an intensity of 5 on a scale of 10. All participants completed 16 training sessions divided into 8 weeks.

### 2.3. Outcomes Measures

The following outcomes were measured before and after 8 weeks of intervention.

#### 2.3.1. Cumberland Ankle Instability Tool (CAIT)

Participants completed the Spanish version of the CAIT [18], a valid and reliable instrument for measuring the severity of CAI. CAIT is a 9-item subjective questionnaire ranging from 0 (severe instability) to 30 (normal stability) [19]. A score equal to or less than 24 determines the presence of CAI [19].

#### 2.3.2. Foot and Ankle Ability Measure (FAAM)

We used FAAM scales for estimating self-reported ankle function. There are two subscales: FAAM-activities of daily living (ADL) and FAAM-sport (S), assessing self-reported ankle functions separately during daily and sports activities. FAAM is a valid outcome instrument for detecting self-reported functional deficits in patients with CAI [20].

#### 2.3.3. Star Excursion Balance Test (SEBT)

The premise of using SEBT is to determine if, while standing on the injured limb to maintain stability, a deficit is produced in the reaching distances, indicating a deficiency in postural control or balance that might be associated with the pathologic condition in the stance limb [21].

The SEBT is valid and useful in demonstrating outcomes from neuromuscular and strengthening exercise interventions [21]. In our measures, we used the three directional variants of SEBT. In this test, the participant had to reach the maximum distance in three directions: anterior (A), posteromedial (PM), and posterolateral (PL) [21].

#### 2.3.4. Weight Bearing Lunge Test (WBLT)

There is strong evidence for good inter- and intra-clinician reliability of the WBLT to assess the Dorsiflexion Range Of Motion (DFROM) [22,23]. The WBLT involves a patient standing in a tandem stance and performing a forward lunge. During this task, the involved foot remains firmly planted on the ground as the tibia is progressed over the talus into maximum dorsiflexion. A variety of measurement techniques can then be used to quantify DFROM. The most common DFROM quantification technique involved the measurement of the distance of the great toe to the wall using a simple tape measure [22]. While performing the WBLT, the clinician controls the position of the feet, the knees, and the pelvis, to check that the test is executed correctly [23].

### 2.4. Statistical Analyses

All analyses were performed using IBM SPSS 24.0 statistical software (IBM Corp, Armonk, NY, USA). The Shapiro–Wilk test was employed to confirm normal distributions of continuous variables (*n* < 50). One-way analysis of variance (ANOVA) tests for continuous variables (if the hypothesis of normality was verified) and Kruskal–Wallis tests for categorical variables (in the absence of normality) were performed to examine baseline differences between three groups.

A one-way ANOVA was applied to all variables, both at the beginning and at the end of treatment. The objective is to detect if there are significant differences between the three groups in which we have classified the individuals for the different variables. A post hoc study was performed in those cases in which the one-way ANOVA is significant. In order to apply the appropriate method of multiple comparisons, first of all, we verified whether the hypothesis of homoscedasticity (equal variances) was verified using the Levene test. If this hypothesis was verified, we used the HSD Tukey method. Otherwise, it was necessary to apply the Games–Howell test.

In order to assess within and between groups’ effect sizes, Cohen’s d effect sizes were calculated. The strength of effects was interpreted as weak (≤0.2), small (0.2 to 0.5), moderate (0.5 to 0.8), and large (>0.8) according to Cohen’s benchmarks [24]. Alpha level was set a prior at *p* < 0.05.

A required number of 21 participants with CAI per group was estimated to ensure a power of 0.80 at a significance level of 95% based on a minimal detectable change of 4.28 cm in the posterolateral direction of SEBT [25], but 24 participants per group were enrolled in the study due to a possible drop-out rate of 15%.

## 3. Results

**Figure 3:**
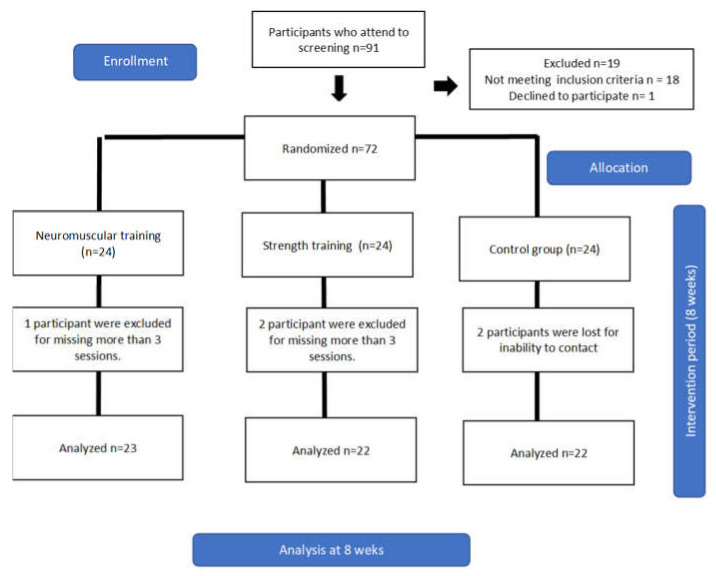
Flow chart of the study design and participants’ follow-up through the trial.

A total of 67 participants were enrolled in the study (Figure 3). No group differed in any of the baseline measures (Table 1), indicating that groups were similar in their demographic and clinical characteristics, except in the anterior direction of the SEBT variable.

As shown in Table 2, the existence of significant differences between the moments before and after the treatment, each variable, and within each group was studied. It can be observed that in the NG and SG groups, there are significant differences between the mean score of the variables at the pre and post-intervention in all variables. In addition, in all cases, observing the value of the CI (values do not cross zero), we concluded that the mean score of the studied variables is higher post-intervention than at baseline.

As shown in Table 3, between-groups differences at the end of the study in the variables SEBT_PM (*p* = 0.000), SEBT_PL (*p* = 0.003), FAAM-ADL (*p* = 0.001) and FAAM-SPORT (*p* = 0.000), were observed.

Significant differences post-intervention were detected between the neuromuscular group and the control group (*p* = 0.001) and between the strength group and the control group (*p* = 0.000). The 95% CI for NG vs. CG is (2355, 10.282), indicating that the mean SEBT-PM score in the neuromuscular group is higher than that of the control group, and the 95% CI for SG vs. CG is (3619, 11.644), indicating that the mean SEBT-PM score in the strength group is higher than that of the control group. Regarding post-intervention scores in SEBT-PL, significant differences were detected between the neuromuscular group and the control group (*p* = 0.031) and between the strength group and the control group (*p* = 0.003). The 95% CI for NG vs. CG is (0.298, 7788), indicating that the mean SEBT-PL score in the neuromuscular group is higher than that in the control group. The 95% CI for SG vs. CG is (0.4292, 1.6914), indicating that the mean SEBT-PL score in the strength group is higher than that of the control group. In the FAAM-ADL, significant differences were detected between the neuromuscular group and the control group (*p* = 0.004) and between the strength group and the control group (*p* = 0.003). The 95% CI for NG vs. CG is (2.74, 16.46), and (2.84, 16.71) in SG vs. CG indicating mean scores in both intervention groups are higher than the control group. In the FAAM-SPORT, significant differences were detected between the neuromuscular group and the control group (*p* = 0.001) as well as the strength training group and the control group (*p* = 0.000) with a 95% CI for NG vs. CG of (4.29, 18.05), indicating higher scores in the NG, and 95% CI for SG vs. CG (6.72, 20.64), indicating in both cases higher scores in comparison with CG.

## 4. Discussion

The present study aimed to determine the influence of neuromuscular training and strength training on functional status, subjective feeling of instability, dynamic balance, and ankle DFROM in patients with CAI. After the literature review, there was no consensus about the superiority of one intervention over another in this population group, and there was heterogeneity in the study population; thus, the objective of the study was to contribute to a better understanding of the management of CAI [10].

Both neuromuscular training and strength training groups improved all variables, with no differences between groups on dynamic balance, functional status, subjective feeling of instability, and ankle DFROM. In contrast with previous research [25,26,27,28,29], the control group improved dynamic balance and CAIT despite not receiving treatment.

The subjective feeling of instability is possibly the most common characteristic in those with CAI, and the influence on functional status and quality of life has been widely addressed [4,18,26]. No statistically significant differences were observed between the two intervention groups, which is similar to results reported by previous research [27] and is in contrast with other studies where the neuromuscular training group was superior to strength training in CAIT scores [9,10]. However, it is possible to identify better improvements on NG in comparison with SG with an effect size of *d* = 0.23. Wright et al. [26] described that Minimal Detectable Change (MDC) and Minimal Clinically Important Difference (MCID) should exceed three points in CAIT to be relevant. Both intervention groups exceeded the MCID with a slight but not statistical superiority of NG vs. SG Cohen’s d (95%) = 0.7808 (0.1745, 1.3872). Final CAIT scores did not reach the cut-off point of 24 to be considered as patients with self-reported stability. The severity of the patients allocated to these groups may be a plausible explanation for these findings.

Self-reported physical function is reduced in subjects with CAI and could be measured with FAAM-ADL and FAAM-SPORT to determine the influence of CAI in daily living activities as well as sport-related activities [20]. Kim et al. [28] found improvements in self-reported function in balance training and hop-stabilization tasks, and Ardakani et al. [29] obtained similar results in an intervention consisting of hop-stabilization training in both FAAM-ADL and FAAM-SPORT subscales. These improvements agree with the obtained results where the NG group showed greater within-group change scores than SG and CG. This suggests that NG intervention could be recommended due to the complex etiology of CAI vs. a more isolating intervention. Regarding SG, obtained results in FAAM-ADL and FAAM-SPORT agree with the described results of Hall et al. [11], with a similar strength-based intervention. Nevertheless, there is no consensus regarding the effectiveness of strength training in CAI patients in the management of FAAM-ADL and FAAM-SPORT outcomes, as reported in a recent systematic review [9] whose authors suggested that further investigation is needed.

The obtained results in this manuscript showed that both NG and SG had improvements after intervention on dynamic balance when measured with SEBT in all directions. Previous studies reported that neuromuscular training improved dynamic balance when compared with baseline, and it could be an effective intervention for secondary prevention for recurrent ankle sprain in sporting populations with CAI [25]. Our results agree with the reported data by Docherty et al. [30], who found that a strengthening program increases strength, inversion joint position sense (JPS), dorsiflexion JPS, and plantar flexion JPS in subjects with functionally unstable ankles. The stimulus provided to mechanoreceptors placed on the myotendinous junction due to the produced force during training may play an important role in proprioception deficit and somatosensory pathways and thus could enhance dynamic balance in patients with CAI [13,14]. In this regard, patients were instructed to focus on controlled movement while performing both strength and balance training to avoid compensatory movements and pain. This control could be related to a sensory reweighting with an important influence in feedback and feedforward mechanism and thus the observed improvement in the participants. In contrast, Hall et al., 2015 [15] showed that strength training could not be effective for improving dynamic balance in individuals with CAI when compared with the control group, which is partially in accordance with our results on anterior and postero-lateral directions. An isolating strength training intervention could be beneficial to promote some muscle groups’ activity, such as peroneus and tibialis, whose coactivation is very important to ankle sprain prevention and functional status in CAI patients. Nevertheless, the dynamic balance depends not only on a muscular group force and required global coordination of the whole body, including core, hip, or knee stabilizers, and especially the feedforward mechanism due to the training protocol. Different outcomes were obtained by Luan et al. [9], who found that balance training could be superior to strength training and no training on SEBT. Our data showed between-group differences in SEBT only when comparing SG and CG on the PM direction of SEBT. These results could be explained by taking into account the observed improvement in the CG, which could be related to the characteristics of the population (active population). Regarding postural control, the inclusion of visual variables as well as the implementation of more functional measurements such as timed up and go test would provide valuable information regarding the effectiveness of both interventions and are suggested to be addressed in future research [31,32].

Reduced DFROM is a frequent characteristic in individuals with CAI [2,4,11]. Decreased or asymmetries on this variable appears to be a risk factor for the development of other injuries such as knee osteoarthritis or ACL injury [2]. Previous studies found improvements in DFROM with neuromuscular training [12]. In our study, there were improvements in DFROM when comparing baseline data in NG and SG, but the change was not enough to be considered statistically significant in comparison with CG. Decreased DFROM has been stated to be correlated with anterior and posterolateral reach directions of the SEBT, but not with PM direction [11], and this could be related to our improvements in PM direction of SEBT in SG because we found differences between groups just in this variable.

## 5. Limitations

- It was not possible to control the daily life activities of participants, and our study population was physically active, which could influence obtained outcomes, especially on CG, because of their usual physical activities. Further research regarding the effects of different CAI-based interventions in active populations is required;
- Target intensity control to calculate the training volume for each patient is difficult;
- Influence of the patient’s previous beliefs and expectations about CAI and the treatment usually received and how popular belief may influence satisfaction with the treatment received, and the results of the variables studied;
- Monitorization of ankle joint muscle strength could provide additional information.

## 6. Conclusions

Our results suggest there are improvements in NG and SG when compared with baseline, obtaining significant differences regarding CG on functional status and posteromedial direction on dynamic balance in SG patients. Although some differences were observed between the intervention groups, it is not possible to establish a relationship of superiority between any of them in an active population with CAI.

## Figures and Tables

**Table 1:** Descriptive summary of baseline measures.

Variables		NG (*n* = 23)	SG (*n* = 22)	CG (*n* = 22)	*p*-Value *
**Demographics**					
Age (yrs)		27.1 ± 5.8	29.5 ± 10.7	27.4 ± 8.3	0.860
	18–30	8 (34.72%)	7 (31.81%)	6 (27.27%)	0.724
N (%)	30–40	9 (39.13%)	12 (54.54%)	10 (45.45%)	
	40–50	4 (17.39%)	2 (9.09%)	3 (13.63%)	
	50–60	2 (8.96%)	1 (4.54%)	3 (13.63%)	
Height (cm)		173.7 ± 7.1	171.8 ± 8.7	170.5 ± 7.9	0.402
Gender: n (%)	Male	14 (60.9%)	11 (50.0%)	16 (72.7%)	0.302
	Female	9 (39.1%)	11 (50.0%)	6 (27.3%)	
Weight (kg)		70.3 ± 8.8	71.2 ± 9.4	68.9 ± 9.2	0.692
Affected ankle: n (%)	Left	10 (43.5%)	12 (54.5%)	8 (36.4%)	0.474
	Right	13 (56.5%)	10 (45.5%)	14 (63.6%)	
**Outcomes**					
DFROM (cm)		6.9 ± 3.0	7.9 ± 2.8	7.6 ± 2.4	0.369
SEBT_Ant (%)		78.1 ± 5.2	77.9 ± 4.4	74.9 ± 3.1	0.032 *
SEBT_PM (%)		82.1 ± 4.9	84.0 ± 5.7	83.9 ± 3.9	0.359
SEBT_PL (%)		79.5 ± 5.9	82.3 ± 7.4	83.8 ± 4.8	0.056
CAIT		14.1 ± 5.2	13.9 ± 4.7	15.7 ± 5.1	0.172
FAAM_ADL		74.6 ± 8.7	76.5 ± 7.4	74.1 ± 9.1	0.615
FAAM_SPORT		67.8 ± 6.4	68.0 ± 6.8	69.1 ± 5.7	0.715

Data are given as means ± standard deviations for continuous variables and frequencies/percentages for categorical variables. Abbreviation: NG, Neuromuscular Group; SG, Strength Group; CG, Control Group; DFROM, Dorsiflexion Range of Motion; SEBT, Star Excursion Balance Test; Ant, anterior; PM, posteromedial; PL, postero-lateral; CAIT, Cumberland Ankle Instability Tool ranging from 0 (severe instability) to 30 (normal stability); FAAM_ADL, Foot and Ankle Ability Measure in Activities of Daily Life; FAAM_SPORT, Foot and Ankle Ability Measure in Sports activities. *p*-value is associated with either one-way analysis of variance test (ANOVA) or with Kruskal–Wallis (depending on whether or not the normality hypothesis is verified) for continuous variables or Pearson’s χ^2^ for categorical variables. * The CG significantly differed from either the NG or SG group.

**Table 2:** Within-Group Change Scores Between Pre- and Postintervention Measurements.

**Group**						
	**NG (*n* = 23)**			**SG (*n* = 22)**		
	**Baseline**	**End of Intervention**	**Within-Group Change (95%CI)**	**Baseline**	**End of Intervention**	**Within-Group Change (95%CI)**
DFROM	6.857 ± 2.969	8.330 ± 2.724	1.4739 (0.712, 2.235) *	7.882 ± 2.814	9.268 ± 2.860	1.3864 (0.803, 1.969) *
SEBT_ANT	78.126 ± 5.154	85.970 ± 6.520	7.8435 (6.305, 9.381) *	77.932 ± 4.399	86.241 ± 4.326	8.3091 (7.206, 9.412) *
SEBT_PM	82.139 ± 4.938	89.887 ± 5.354	7.7478 (4.907, 10.588) *	84.000 ± 5.740	91.205 ± 5.363	7.2045 (4.936, 9.472) *
SEBT_PL	79.513 ± 5.889	88.161 ± 5.378	8.6478 (6.013, 11.282) *	82.282 ± 7.357	89.586 ± 4.877	7.2045 (4.739, 9.670) *
CAIT	14.13 ± 5.216	20.74 ± 5.172	6.609 (4.281, 8.936) *	13.91 ± 4.740	20.50 ± 4.585	6.591 (4.88, 8.293) *
FAAM_ADL	74.57 ± 8.649	84.78 ± 10.278	10.217 (6.678, 13.757) *	76.45 ± 7.398	84.95 ± 7.524	8.500 (4.93, 12.070) *
FAAM_SPORT	67.83 ± 6.365	83.26 ± 9.724	15.435 (11.638, 19.231) *	68.00 ± 6.782	85.77 ± 7.197	17.773 (15.325, 20.220) *
**Group**						
**CG (*n* = 22)**						
	**Baseline**		**End of Intervention**		**Within-Group Change (95%CI)**	
DFROM	7600 ± 2406		8086 ± 2.723		0.486 (−0.111, 1.084)	
SEBT_ANT	74.909 ± 3.102		83.314 ± 6.374		8.404 (5.976, 10.832) *	
SEBT_PM	83.923 ± 3.864		83.568 ± 5.890		−0,354 (−2.887, 2.178)	
SEBT_PL	83.745 ± 4.747		84.118 ± 5.422		0.372 (−1.184, 1.930)	
CAIT	15.73 ± 5.091		17.59 ± 6.045		1.864 (0.535, 3.192) *	
FAAM_ADL	74.09 ± 9.050		74.18 ± 10.613		1.091 (−2.106, 4,288)	
FAAM_SPORT	69.05 ± 5.652		72.09 ± 11.447		3.045 (−1.072, 7.163)	

Abbreviation: NG, Neuromuscular Group; SG, Strength Group; CG, Control Group; DFROM, Dorsiflexion Range of Motion; SEBT, Star Excursion Balance Test; Ant, anterior; PM, posteromedial; PL, postero-lateral; CAIT, Cumberland Ankle Instability Tool; FAAM_ADL, Foot and Ankle Ability Measure in Activities of Daily Life; FAAM_SPORT, Foot and Ankle Ability Measure in Sports activities. Data are given as mean ± SD; Statistical Differences *.

**Table 3:** Between-Groups Effect Sizes.

	NG vs. SG		NG vs. CG		SG vs. CG	
	Between-Group Change (95% CI)	Cohen d (95%)	Between-Group Change (95% CI)	Cohen d (95%)	Between-Group Change (95% CI)	Cohen d (95%)
DFROM	−0.982 (−2880, 0.917)	−1.7555 (−2.4434, −1.0676)	−0.250 (−2.149, 1649)	−0.4469 (−1.0389, 0.1448)	0.732 (−1188, 2652)	1.2933 (0.6435, 1.9431)
SEBT_Ant	−0.039 (−3418, 3341)	−0.0001 (−0.5845, 0.5844)	2.936 (−0.443, 6.316)	0.0042 (−0.5803, 0.5887)	2.975 (−0.442, 6392)	2.9543 (2.0998, 3.8089)
SEBT_PM	−1589 (−4.697, 1518)	−1.736 (−2.4218, −1.0503)	2.268 (−0.840, 5.375)	2.4778 (1.7009, 3.2548)	3.857 (0.715, 6998) *	4.1652 (3.1133, 5.2172)
SEBT_PL	−2147 (−5760, 1466)	−2.0164 (−2.7341, −1.2986)	−0.095 (−3708, 3.518)	−0.0892 (−0.674, 0.4956)	2.052 (−1601, 5705)	1.9053 (1.1928, 2.6178)
CAIT	0.23 (−3.14, 3.60)	0.2314 (−0.355, 0.8179)	0.78 (−2.60, 415)	0.7808 (0.1745, 1.3872)	0.55 (−2.86, 3.96)	0.5433 (−0.0585, 1145)
FAAM_ADL	−1.03 (−6.84, 4.78)	−0.6027 (−1.2003,−0.0051)	5.04 (−0.77, 10.84)	2.9451 (2.1014, 3.7888)	6.07 (0.20, 11.94) *	3.5081 (2.5666, 4.4496)
FAAM_SPORT	−1.34 (−6.39, 3.71)	−0.9024 (−1.5159, −0.2889)	4.98 (−0.07, 10.03)	3.3429 (2.4382, 4.2477)	6.32 (1.21, 11.42) *	4.198 (3.1404, 5.2556)

Abbreviation: NG, Neuromuscular Group; SG, Strength Group; CG, Control Group; DFROM, Dorsiflexion Range of Motion; SEBT, Star Excursion Balance Test; Ant, anterior; PM, posteromedial; PL, postero-lateral; CAIT, Cumberland Ankle Instability Tool; FAAM_ADL, Foot and Ankle Ability Measure in Activities of Daily Life; FAAM_SPORT, Foot and Ankle Ability Measure in Sports activities.; Between-groups change scores were calculated using postintervention measurements between 2 of the 3 groups, with positive values indicating that the intervention listed first was more effective.;Cohen d estimates of effect sizes were computed by dividing the change scores by the pooled standard deviations; Statistical Differences (*).

## Data Availability

The data are not publicly available due to data protection local law.
